# Ecto-5' -Nucleotidase CD73 (NT5E), vitamin D receptor and FGF23 gene polymorphisms may play a role in the development of calcific uremic arteriolopathy in dialysis patients – Data from the German Calciphylaxis Registry

**DOI:** 10.1371/journal.pone.0172407

**Published:** 2017-02-17

**Authors:** Hansjörg Rothe, Vincent Brandenburg, Margot Haun, Barbara Kollerits, Florian Kronenberg, Markus Ketteler, Christoph Wanner

**Affiliations:** 1 Klinikum Coburg, Coburg, Germany; 2 Department of Medicine, Division of Nephrology, Julius-Maximilians-Universität Würzburg, Würzburg, Germany; 3 Department of Cardiology, RWTH University of Aachen, Aachen, Germany; 4 Division for Genetic Epidemiology, Medical University of Innsbruck, Innsbruck, Austria; Universidade de Sao Paulo, BRAZIL

## Abstract

**Introduction:**

Calciphylaxis/calcific uremic arteriolopathy affects mainly end-stage kidney disease patients but is also associated with malignant disorders such as myeloma, melanoma and breast cancer. Genetic risk factors of calciphylaxis have never been studied before.

**Methods:**

We investigated 10 target genes using a tagging SNP approach: the genes encoding CD73/ ecto-5'-nucleotidase (purinergic pathway), Matrix Gla protein, Fetuin A, Bone Gla protein, VKORC1 (all related to intrinsic calcification inhibition), calcium-sensing receptor, FGF23, Klotho, vitamin D receptor, stanniocalcin 1 (all related to CKD-MBD). 144 dialysis patients from the German calciphylaxis registry were compared with 370 dialysis patients without history of CUA. Genotyping was performed using iPLEX Gold MassARRAY(Sequenom, San Diego, USA), KASP genotyping chemistry (LGC, Teddington, Middlesex, UK) or sequencing. Statistical analysis comprised logistic regression analysis with adjustment for age and sex.

**Results:**

165 SNPs were finally analyzed and 6 SNPs were associated with higher probability for calciphylaxis (OR>1) in our cohort. Nine SNPs of three genes (CD73, FGF23 and Vitamin D receptor) reached nominal significance (p< 0.05), but did not reach statistical significance after correction for multiple testing. Of the CD73 gene, rs4431401 (OR = 1.71, 95%CI 1.08–2.17, p = 0.023) and rs9444348 (OR = 1.48, 95% CI 1.11–1.97, p = 0.008) were associated with a higher probability for CUA. Of the FGF23 and VDR genes, rs7310492, rs11063118, rs13312747 and rs17882106 were associated with a higher probability for CUA.

**Conclusion:**

Polymorphisms in the genes encoding CD73, vitamin D receptor and FGF23 may play a role in calciphylaxis development. Although our study is the largest genetic study on calciphylaxis, it is limited by the low sample sizes. It therefore requires replication in other cohorts if available.

## Introduction

Calcific uremic arteriolopathy (CUA) or calciphylaxis is a rare condition of accelerated calcification of skin arterioles[1] (diameter around 100μ) which mainly develops in end-stage renal disease patients (ESRD) patients. It does also occur in patients with malignant diseases (such as myeloma[2], melanoma[3] and breast cancer[4]) and normal renal function[5]. It reduces quality of life considerably and still carries a one-year-mortality risk of approximately 50%, mainly due to superimposed sepsis. [1] The yearly incidence is <1% in patients on maintenance dialysis[1].

The diagnosis is made clinically in the presence of progressive, painful, retiform violaceous (later black/necrotic) skin lesions, which develop into large retiform ulcerations with thick eschar due to microthrombi formation and tissue necrosis.[1] Skin biopsies may sometimes clarify the diagnosis, but additional invasive procedures should generally be used with great caution, whereas the recently proposed method of showing calcified material in debrided tissue by microcomputed tomography (Raman spectroscopy) is not usually available.[6] Particularly in proximal lesions large deep fat tissue ulcerations may develop, carrying an especially poor prognosis.

Although chronic kidney disease is the most important clinical risk factor, followed by malignancies, CUA does also occur in association with normal kidney function and liver cirrhosis.[7] Other risk factors are female sex[1], obesity[1], thrombophilia syndromes such as Protein S or C deficiency[8], treatment with vitamin K antagonists[9] and/or corticosteroids and low albumin levels.[1]

Regarding the pathogenesis of CUA, the scientific community had pursued the hypothesis of the calcification process as a continuum, from vascular calcification in general to extra-skeletal osteogenesis and CUA [10], over the last two decades. However, recent surveys and registry data suggest that conditions associated with high calcium-phosphorus product (primary and secondary hyperparathyroidism) play only a secondary role for CUA. [11] Therefore it has been argued that extra-skeletal osteogenesis and CKD-MDB might be regarded as a sensitization, which after a latency period is followed by an acute trigger event. This etiology theory is quite close to the one that had already been proposed by Hans Selye[12] who coined the term calciphylaxis in 1965. The fact that only a minority of patients with the same risk profile will develop the unique picture of CUA is reflected better by this two-step hypothesis as compared with the continuum one.

The hypothesis was developed further with the discovery of autosomal recessive CD73 deficiency[13], a calcification syndrome causing a phenotype which resembles the classical picture of medial artery calcification[14]. The purinergic signalling pathway, which the ecto-5'-nucleotidase CD73, also referred to as NT5E, belongs to, emerged as a possible mechanism for this acute CUA triggering event. Moreover, CD73 is a key regulatory molecule of cancer cell proliferation, migration and invasion in vitro, tumor angiogenesis, and tumor immune escape in vivo[15].

We therefore designed a case-control study to look into genetic risk profiles of CUA in the German calciphylaxis registry patients based on a target gene approach and included the CD73 (or NT5E) gene in the list, besides genes of the CKD-MBD complex and genes related to intrinsic calcification inhibitors.

## Results

All statistical analyses were adjusted for sex and age. There were significantly more female patients in the CUA group than in the control group (Chi-squared test, p<0.001). There were only slight differences in age between the groups (Mann-Whitney-U test)(see Table 1).

**Table 1:** Demographics.

Cohort	Calciphylaxis registry patients		Control group	
	• (n = 144)		• (n = 370)	
	female	male	female	male
**N total**	83 (58%)	61 (42%)	146 (39%)	224 (61%)
**Age (years)**	68±13	65±12	71±13	68±14
	• (63; 70; 77)	• (59; 67; 73)	• (65; 75; 80)	• (58; 73; 80)

Genotyping results were available from 172 out of 207 selected SNPs(for all results see supporting information S1 File, file calziphylaxie_final.xlsx). Seven SNPs were excluded for too small numbers (either zero and/or n = 1) of heterozygote or homozygous carries of the minor allele. Thus, finally 165 SNPs were included in this analysis. Calculation of λ revealed an additive mode of inheritance as the most appropriate genetic model for most of the SNPs except for two where a dominant model was selected (rs4431401 and rs12812339, see Table 2). Therefore, all further analyses were based on the additive and dominant inheritance assumption, respectively. Genotype frequencies of all nominal significant SNPs were in Hardy-Weinberg-Equilibrium (HWE) in the CUA cases and controls (p >0.05). Six SNPs from 3 of the 10 genes tested (encoding CD73, FGF23 and Vitamin D receptor) were associated with a higher probability for calciphylaxis in our cohort (OR>1). However these SNPs only reached nominal significance (p< 0.05) (see Table 2). No SNP reached significance after Bonferroni correction for multiple testing. All SNPs with nominal significance see Table 2.

**Table 2:** Single nucleotide polymorphisms (SNPs) that were nominal significantly different in frequency between patients and controls. Data are adjusted for age and sex.

Gene encoding	SNP	N total	Risk allele^$^	Reference allele^$^	OR^§^	95% confidence interval	P value
**CD73**	Rs9444348	512	G	A	1.48	1.11–1.97	0.008
**FGF23**	Rs12812339	503	C	A	0.58	0.38–0.87	0.009
**FGF23**	Rs7310492	510	A	T	1.49	1.06–2.09	0.021
**CD73**	Rs4431401	512	T	C	1.71	1.08–2.71	0.023
**VDR**	Rs10783223	512	G	T	0.73	0.55–0.96	0.025
**VDR**	Rs17882106	496	T	C	1.65	1.06–2.56	0.026
**FGF23**	Rs6489536	511	C	G	0.70	0.51–0.96	0.026
**FGF23**	Rs11063118	511	C	T	1.41	1.03–1.92	0.032
**FGF23**	Rs13312747	497	C	G	1.50	1.01–2.25	0.047

§ effect estimate refers to the risk allele.

$ based on total group (cases and controls).

## Discussion

There are two different scenarios where genetic association studies may be helpful in rare diseases: 1. If a causative genetic abnormality is suspected genome wide association studies may identify new polymorphisms or mutations. In this scenario an association with SNP´s that are prevalent in the healthy population is unlikely. 2. If there is no reason to assume a causative genetic abnormality but the underlying pathomechanisms are unknown, association with polymorphisms altering certain gene functions may produce new hypotheses regarding the pathophysiology of the disease. In this scenario SNP´s that are prevalent in the healthy population are helpful.

CUA shows no family clustering, the rationale to perform our study falls under the second scenario. The present study provides these major results: of the intronic region of the CD73 gene, rs4431401 (and rs9444348 were associated with a higher probability of CUA in our cohort. Rs4431401 has been found to modulate expression of small nucleolar RNA host gene 5 (SNHG5) in a genome wide identification study of expression quantitative trait loci (eQTLs) in human heart.[16] Small nucleolar RNAs belong to the long non-coding RNAs (lncRNAs) and regulate rRNA modification mainly by altering the methylation of fibrillarin.[17] The RNA serum level of SNHG5 in particular has been proposed as a new tumor marker of malignant melanoma[18]- a condition which is often associated with calcification[19,20, 21, 22] even in patients with normal kidney function.

Rs4431401 is also in strong linkage disequilibrium with promoter flanking region SNP rs9344530 as well as CTCF binding site SNPs rs117478631 and rs9362223 in European populations (1000genomes phase 3 data). Rs9444348 is situated in the promoter flanking region of the CD73 gene, which could result in an altered transcriptional regulation of its gene product. CD73 plays a role of fine-tuning the effects within the purinergic signalling pathway. Adenosine, which is mainly produced by CD73 locally, attenuates both the pro-inflammatory [23] and the pain-stimulatory response[24] initiated by eATP. CD73 knock-out mice seem to cope better than patients with inactivating CD73 mutations as far as calcification is concerned. However, just like many dialysis patients, they develop juxta-articular joint-capsule mineralization.[25] A possible mechanism how CD73 inactivation could lead to calcification is proposed by Markello et al: adenosine also inhibits tissue-neutral alkaline phosphatase, which in turn degrades the calcification inhibitor pyrophosphate, produced by ENPP1.[26]

Of the vitamin D receptor (VDR) gene, rs17882106 is associated with a higher probability of CUA in our cohort. Significance for this SNP disappeared when CUA patients not requiring dialysis were excluded from analysis. This SNP lies within a binding region for the 11-zinc-finger protein or CTCF, a transcriptional repressor. Again this could affect transcriptional regulation of the receptor molecule. Activated vitamin D is one of the factors identified to be involved in calciphylaxis already several decades ago.[27] Finally, of the fibroblast growth factor FGF23 gene, rs7310492, rs11063118 and rs13312747 are associated with a higher probability for CUA in our cohort. However, none of these FGF23 gene SNPs are associated with regulatory features, although rs11063118 has been found to be associated with an increased risk of prostate cancer in a Korean population.[28]

Although our study investigated the biggest CUA cohort studied so far, it is still limited by the rather low patient numbers. Therefore a replication of our study in another comparable patient cohort would be desirable. In rare conditions such as calciphylaxis even the biggest registry will produce comparatively low patient numbers. While this obviously reduces the power of the study, the same is true for the number of target genes: adding more genes to the list, would have increased the number of tagging SNPs resulting in even bigger type I error bias due to multiple testing.[29] Therefore, no other gene of the purinergic pathway was chosen in addition to CD73, although ENPP1,[30] for-instance, is also well known to be associated with vascular calcification.

One reason to perform this study was the hope that a better understanding of CUA pathomechanisms might eventually lead to new diagnostic and prognostic laboratory parameters. At present the diagnosis is made clinically. Histology may be helpful but the skin biopsy procedure may in itself worsen the condition. Due to the severe condition the patients are usually in, a referral to a tertiary center to provide independent review of the diagnosis is impossible in most cases. This raises the question of bias in our CUA cohort–the proportion of patients with minor stages of CKD not requiring dialysis was relatively high in our CUA cohort (16 out of 144 [11%]) which might be interpreted as a bias towards cases with higher genetic burden. We therefore performed a sensitivity analysis by excluding these 16 cases. This analysis showed no major changes in effect estimates and thus the final conclusion remains unchanged.

Regarding a possible enhancement of our currently rather limited therapy options,[31] focus on the CD73 cascade is promising, since genetic calcification syndromes may be treatable with ENPP1-Fc fusion protein injections, as recently shown in a mouse model. [32] RNAs have also been proposed as therapy targets in clinical patterns related to CUA, for-instance micro RNAs involved in nociceptive circuits.[33]

In summary, polymorphisms of genes encoding CD73, vitamin D receptor and FGF23 may play a role in calciphylaxis development. The results of this genetic risk study seem to reinforce the title of a previous paper published in the journal of the German society of dermatology: “Calciphylaxis. A call for interdisciplinary co-operation”.[34]

## Methods

### Rationale for selecting the genes

The CKD-MBD group of genes had to include the calcium-sensing receptor [35] and vitamin-D receptor[36] genes since drugs affecting these two receptors are well known to have an impact on the clinical course of CUA in many patients. Klotho and FGF23[37] are associated with CKD-MBD development in many ways, while stanniocalcin was identified in a GWAS of renal failure.[38]

The intrinsic calcification group of genes had to include Matrix Gla Protein (MGP) [39] and Fetuin A (AHSG) [40] since deficiency syndromes of both inhibitors are known to cause calcification. Bone Gla Protein (BGLAP) [41] is genetically closely related to MGP, while VCORC1 is known to affect vitamin K[42] dependent MGP and also Anti-Vitamin K therapy which again is an important risk factor for CUA.

### Rationale for selecting the SNP´s

In the tagging SNP approach [43] optimal sets of single nucleotide polymorphisms are derived from genetic databases in order to tag haplotypes across genes. The entire set of genetic variants (including +/- 5kb flanking region), with minor allele frequency >10% in European population (including CEU, FIN, GBR, IBS, TSI) from the International HapMap Project (release #28; http://www.hapmap.org) was included. Tagging SNPs were selected using the Tagger algorithm in Haploview (http://www.broad.mit.edu/mpg/haploview) with a minimum r^2^ of 0.8. This selection resulted in 187 tagging SNPs. Additional 20 SNPs in the CaSR gene were included which have been hypothesized before to alter the conformation of the CaSR molecule and thereby possibly influence calcium-sensing and/or calcimimetic therapy (unpublished data).

### Patients–CUA cohort

The diagnosis of calciphylaxis was made by the attending physician in all cases enrolled into the German calciphylaxis registry. There was no independent review of records. A skin biopsy was performed in 45% of CUA cases[11]. 144 consecutive CUA patients with end-stage kidney disease on maintenance dialysis and clinical calciphylaxis symptoms, from dialysis centers throughout Germany, who had been registered in the German calciphylaxis registry between 2006 and 2014, were included. All patients were white. Due to the usually devastating time-course of CUA a differentiation into less severe or more severe cases was not provided. A differentiation into cases in whom mainly extremities are involved from others with mainly central involvement of the trunk may be possible, but in general such differences reflect different stages in the time-course of the disease rather than true phenotypic differences.

16 out of 144 patients (11%) in the CUA cohort did not require dialysis, whereas all control patients had been on dialysis for at least 6 months.

#### Inclusion criteria

All patients registered by their attending nephrologists as having clinical signs of CUA in the online nationwide German CUA registry, who had signed the consent form and had blood taken for genetic analysis, and who were older than 18 years, were eligible. Exclusion criteria: age < 18 years, not having signed the consent form or any disorder that would interfere with understanding protocol requirements.

### Patients—Control cohort

The ethics review board of Bayerische Landesärztekammer, Munich, Germany had approved the study and its consent form (study number 13080). 370 patients with end-stage kidney disease who never had clinical calciphylaxis, recruited at 5 dialysis centers in the German regions Franken and Thüringen in 2013 and 2014. inclusion criteria: All patients of the five participating centers who had been registered by their attending nephrologists as never having had clinical signs of CUA were eligible, inclusion criteria were otherwise identical to the CUA cohort. Exclusion criteria: Since all CUA registry patients were white, control patients with other ethnic backgrounds had to be excluded, otherwise exclusion criteria were identical to the CUA cohort. Eleven patients were excluded; one patient because of ethnicity (African American), and 10 patients due to missing data (age, gender or signed consent form).

### Genotyping

Genotyping was performed by using iPLEX Gold MassARRAY (Sequenom, San Diego, USA), KASP genotyping chemistry (LGC, Teddington, Middlesex, UK) or sequencing, respectively. Genotyping results were available from 172 out of 207 selected SNPs. The different count is due to technical problems within the genotyping and the exclusion of individual SNPs from genotyping, in case the SNPs were tagging only itself.

### Statistical analysis

In the analyses the multiple testing problem was accounted for by performing Bonferroni correction assuming 172 independent tests (SNPs) resulting in a new level of significance of 0.0003 (p = 0.05/172). Genotype and allele frequencies for CUA cases and controls were estimated and a test of Hardy-Weinberg equilibrium (HWE) was performed. Beta estimates (log odds ratios) were calculated using a logistic regression model without assuming a genetic mode of inheritance (unconstrained model). The choice of the genetic mode of inheritance was based on the beta estimates (log odds ratios) of the unconstrained inheritance model and their corresponding quotient λ = heterozygote carriers / homozygote carriers of the minor allele as described previously.[44] For a recessive model, this quotient λ is around 0, whereas λ is about 0.5 for an additive model and about 1 for a dominant model. Furthermore, age-, and sex-adjusted odds ratios and 95% confidence intervals for each SNP were obtained using logistic regression analysis based on λ and thus the most appropriate genetic model. All statistical analyses were performed with the Statistical Package for the Social Sciences (SPSS) for Windows, version 21.0 (IBM Corp., Armonk, New York, NY, USA) and R for Windows, version 3.1.3 (Vienna, Austria).

## Supporting information

S1 File Calciphylaxie final. (XLSX)
